# Flor yeast immobilization in microbial biocapsules for Sherry wine production: microvinification approach

**DOI:** 10.1007/s11274-023-03713-1

**Published:** 2023-08-05

**Authors:** Noelia Pastor-Vega, Juan Carbonero-Pacheco, Juan Carlos Mauricio, Juan Moreno, Teresa García-Martínez, Nitin Nitin, Minami Ogawa, Rewa Rai, Jaime Moreno-García

**Affiliations:** 1 Department of Agricultural Chemistry, Edaphology and Microbiology, University of Córdoba, Córdoba, 14014 Spain; 2 Department of Food Science and Technology, University of California, Davis, Davis, CA 95616 USA

**Keywords:** Sherry wine, Flor yeast, Flor biofilm, Fortification, Microbial biocapsules, Glycerol consumption pre-acclimatization

## Abstract

Sherry wine is a pale-yellowish dry wine produced in Southern-Spain which features are mainly due to biological aging when the metabolism of biofilm-forming yeasts (flor yeasts) consumes ethanol (and other non-fermentable carbon sources) from a previous alcoholic fermentation, and produces volatile compounds such as acetaldehyde. To start aging and maintain the wine stability, a high alcohol content is required, which is achieved by the previous fermentation or by adding ethanol (fortification). Here, an alternative method is proposed which aims to produce a more economic, distinctive Sherry wine without fortification. For this, a flor yeast has been pre-acclimatized to glycerol consumption against ethanol, and later confined in a fungal-based immobilization system known as “microbial biocapsules”, to facilitate its inoculum. Once aged, the wines produced using biocapsules and free yeasts (the conventional method) exhibited chemical differences in terms of acidity and volatile concentrations. These differences were evaluated positively by a sensory panel. Pre-acclimatization of flor yeasts to glycerol consumption was not successful but when cells were immobilized in fungal pellets, ethanol consumption was lower. We believe that immobilization of flor yeasts in microbial biocapsules is an economic technique that can be used to produce high quality differentiated Sherry wines.

## Key points

• Flor yeasts were immobilized in microbial biocapsules and used for Sherry winemaking.

• Biocapsules decrease ethanol consumption over free flor yeast cells during winemaking.

• Use of biocapsules improves Sherry wine organoleptic properties.

## Introduction

Biological aging is a winemaking microbiological practice required for the production of a special type of wine known as “Sherry”, which is usually produced in the Southern-Spain in areas like Condado de Huelva, Jerez-Xérès-Sherry and Montilla-Moriles. This process is carried out after alcoholic fermentation, in oak casks for over 3 years where a biofilm, known as flor, produced by *Saccharomyces* flor yeast develops on wine-air interphase in a dynamic system called “criaderas and solera” in which the aged wine ready to consume is kept in the solera (Alexandre 2013; Moreno-García et al. 2013; Legras et al. 2016; Ferreiro-González et al. 2019; Sancho-Galán et al. 2019). This system facilitates the maintenance of Sherry wine organoleptic characteristics at their final stage (solera), by replacing the consumed volume with Sherry wine from the next aging level (first criadera) (Valcárcel-Muñoz et al. 2022).

Flor is mostly composed of different *Saccharomyces cerevisiae* strains (Esteve-Zarzoso et al. 2004). However, the recent application of molecular identification tools such as microsatellite typing, Internal Transcribed Spacer (ITS)-metabarcoding and Matrix-Assisted Laser Desorption/Ionization Time of Flight (MALDI-TOF) Mass Spectrometry, has revealed the presence of non*-Saccharomyces* yeasts such as *Pichia manshurica*, *Pichia membranifaciens*, *Wickerhamomyces anomalus*, *Candida guillermondii*, and *Trichosporon asahii* (Ruiz-Muñoz et al. 2020; Carbonero-Pacheco et al. 2021). These species were quantified in low amounts, and seem to not have a significant effect on the wine quality (Carbonero-Pacheco et al. 2021).

The yeast in the flor establishes an oxidative metabolism, in which they can consume non-fermentable carbon sources such as ethanol, glycerol, acetic acid, and/or lactic acid. In this way, acetaldehyde, 1,1-diethoxyethanem 2,3-butanediol, diacetyl, acetoin, fusel alcohols, among others, are released as reaction products, thus conferring a wide range of organoleptic properties to the wine such as ripe to overripe apple, creamy, or roundness (Peinado and Mauricio 2009; Moreno-García et al. 2018b; Sancho-Galán et al. 2019). The increased levels of acetaldehyde and the decreased concentration of ethanol, glycerol, and volatile acidity are the most significant chemical changes in biological aging (Pozo-Bayón et al. 2011). Further, the flor protects wine from oxidation (Mauricio and Ortega 1997; Moreno-García et al. 2018b) while allowing evaporation of ethanol and other compounds (Pozo-Bayón et al. 2011).

Usually, there is a fortification or alcohol addition step before biological aging. The objective of this phase is to increase the alcohol content and biological stability of the wine during the aging process. Nonetheless, this practice is not essential in some warm regions such as Montilla-Moriles (southern Spain), where wines reach > 15% (v/v) ethanol content naturally through alcoholic fermentation. Wines with a low alcohol content that will later be subjected to biological aging need to be added until they reach an alcohol content of over 14% (v/v) (BOJA 2023), which causes increases in imposed taxes and hence increases the price of the final product.

Here, we conducted microvinifications to produce Sherry wine using flor yeasts immobilized in a natural system known as microbial biocapsules. Immobilization systems facilitate yeast handling and recovery and increase protection of cells from stress conditions (physical and/or chemical) (Moreno-García et al. 2018a; López-Menchero et al. 2021). Microbial biocapsules use fungal spherical pellets where the yeast cells are encapsulated and attached to the hyphae. This system is an improvement of a previous technology known as “yeast biocapsules” that have been tested for the production of alcoholic beverages (Peinado et al. 2005, 2006; Puig-Pujol et al. 2013; García-Martínez et al. 2015; López de Lerma et al. 2018; López-Menchero et al. 2021; Liu et al. 2022; Ogawa et al. 2022;). Through its utilization, sensory attributes of some of these beverages have been significantly enhanced. Further, flor yeasts have been pre-acclimatized for glycerol consumption to reduce ethanol consumption with the final goal of avoiding or complementing the fortification step and cheapen the process. We expect to produce a more economic and higher quality wine or at least a differentiated wine, through yeast immobilization on natural supports.

## Materials and methods

### Microorganisms, media and growth conditions

In this study, the strains used were *S. cerevisiae* G1 (ATCC: MYA-2451), a flor yeast used in the biological aging of Sherry-type wines, and the filamentous fungus (ff) *Aspergillus oryzae* UCD 76 − 2, a food-grade, generally recognized as safe (GRAS) ff, from the Agricultural Chemistry, Edaphology and Microbiology Department of the University of Córdoba (Spain) and Phaff Culture Collection at the University of California, Davis (USA); respectively. The yeasts were incubated in 50 mL of YPD (g/L): yeast extract, 10; peptone, 20; and dextrose, 20; in 100 mL Erlenmeyer flasks overnight at 175 rpm and 28ºC. The seed culture was pelleted by centrifugation (5000 rpm, 15 min; Hettich Zentrifugen Rotina 38R, ø 15 cm) and scaled up to 500 mL of YPD in 1000 mL Erlenmeyer flasks overnight at 175 rpm and 28ºC. The ff was cultured on sporulation agar medium (g/L): corn meal agar, 17; yeast extract, 1; glucose, 2; agar, 20; for 7 days at 28 °C.

### Pre-acclimatization to glycerol consumption

Cultured yeast cells were pelleted by centrifugation (7000 rpm, 15 min; Beckman Coulter J2-HS Centrifuge, ø 30 cm) and transferred to a 500 mL YPG medium (g/L): yeast extract, 10; peptone, 20; and glycerol, 30; and cultured for 5 days at 175 rpm and 28ºC. Afterwards, the yeast cells were pelleted and inoculated in glycerol-added wine. This wine is the same base wine used for further biological aging but with a final glycerol concentration of 9 g/L. The acclimatization was performed for 5 days at 175 rpm and 28 ºC. Contamination tests through direct microscopic observation and sample cultures in YPD-agar were conducted after each acclimatization phase.

### Yeast immobilization procedure

Once the pre-acclimatization finished, the yeast cells were pelleted by centrifugation (7000 rpm, 15 min; Beckman Coulter J2-HS Centrifuge, ø 30 cm) and a portion was used to immobilize in microbial biocapsules (MB). To produce MB, ff spores were harvested from the sporulation agar medium into a vessel with sterile distilled (DI) water, vortexed and sonicated for 5 min to obtain a homogeneous spore suspension. Ff spores were inoculated to reach a final population of 1 × 10^6^ spores/mL in a fungal pellet culture medium (FPM) consisting of (g/L): glucose, 60; yeast extract, 3; NaNO_3_, 3; K_2_HPO_4_, 1; MgSO_4_, 0.5; KCl 0.5; and FeSO_4_; 0.01 and adjusted to pH 5.5 with HCl); and cultured for 3 days at 175 rpm, 30 °C; to form the fungal pellets. A 50 mL Falcon tube was filled with sterile DI water and cultured yeast cells and ff pellets were immersed in a ratio of 1:1 wet weight yeast:ff pellet. This suspension was subjected to vacuum infusion (< 0.3 atm pressure) for 1 min using a Bonsenkitchen system (Oakwood, GA, USA), which forces the yeast cells suspension into the ff pellets’ air pockets. To confirm infusion, absorbance at 580 nm was measured in the cell suspension both before and after the vacuum step; a 20% reduction in absorbance was obtained. Ff pellets with encapsulated yeast cells were submerged into a YPD solution and cultivated overnight at 175 rpm, 28 °C. The acquired MB were then rinsed thoroughly in sterile DI water to detach any surface cells that can cause cell leakage during the aging. The MB assembling methodology has been submitted to patent application (Application number: 64/411,843).

### Biological aging conditions

The base wine (BW) used for biological aging was provided by Alvear, a local winery from Montilla-Moriles winemaking region, Córdoba (Spain). It consisted of a white wine obtained from Pedro Ximénez grape variety, stored for 8 months in a clay jar (i.e., “tinaja”), filtered over diatomaceous earth and kept under nitrogen in a stainless steel tank. It is a low alcoholic wine with 13.75% (v/v) ethanol, pH of 3.25, titratable acidity of 5.05 g of tartaric acid/L, volatile acidity of 0.42 g of acetic acid/L, and free SO_2_ of < 6 mg/L.

For biological aging microvinifications, 250 mL BW in sterile 250 mL Erlenmeyer flasks (air:wine proportion similar to wine barrels) were inoculated to reach a final concentration of 1.2 × 10^6^ cells/mL, in different formats: microbial biocapsules (MB), suspended or free yeast cells (FY) and non-pre-acclimatized free yeast cells (NPFY) to analyze the impact of glycerol pre-acclimatization of yeasts. All flasks were covered with hydrophobic cotton and aged at 21ºC and a relative humidity (RH) of 70% for 3 months without agitation. Biological aging was monitored through the observation of flor formation and changes in ethanol and glycerol concentration after the first month of inoculation through enzymatic kits (Boehringer Manheim/R-Biopharm, Darmstadt, Germany and Sigma-Aldritch, St. Louis, MO, USA; respectively). Contamination tests were conducted before and after biological aging.

### Measurement of enological parameters

After aging, the sample supernatant was separated from the solid particles through centrifugation (7500 rpm for 15 min). Ethanol content, titratable and volatile acidity, and pH were quantified using the methods recommended by the International Organization of Vine and Wine (OIV). Glycerol was measured using the glycerol assay kits (R-Biopharm, St.Louis, USA). To quantify the effect of basal evaporation on the reduction of ethanol content, a condition was established with identical biological conditions (21ºC, 70% RH, 3 months, no stirring) but without the inoculation of flor yeast. A decrease of 2.55 ± 0.67% (v/v) ethanol was reported due to basal evaporation. It should be noted that ethanol evaporation rates are higher when aging is carried in Erlenmeyer flasks covered with a hydrophobic cotton versus the oak barrels (Moreno-García et al. 2013, 2014; Wollan et al. 2016).

### Quantification of major aroma compounds and polyols

The major volatile compounds are those found > 10 mg/L that influence the wine organoleptic characteristics (aroma and flavor). To quantify them, 10 mL of wine sample was mixed with 1 mL of internal standard solution (4-methyl-2-pentanol) and 0.2 g of calcium carbonate. The mixture was then placed in an ultrasound bath for 30 s, and centrifuged for 10 min at 5000 rpm and 2ºC. After the centrifugation, the supernatant was separated and 1 μL was injected in a Gas Chromatograph (GC) Agilent 6890 (Palo Alto, California, USA) with a fused silica capillary column (60 m, 0.25 mm diameter and 0.4 μm film) coupled to a Flame Ionization Detector (FID). The oven was set to 45ºC during the first 15 min, and the temperature increased to 190ºC at a rate of 4 ºC/min for 35 min. The injector and detector were programmed to 270ºC and 300ºC, respectively. The chromatograph was set so that only a tenth of the sample was able to get through the column. Helium was used as the carrying gas. The flow speed was 0.7 mL/min for the first 16 min, and then it was increased to 1.1 mL/min at a rate of 0.2 mL maintained for 52 min. The chromatographic peaks were assigned to a specific compound by its retention time (RT) and by the addition of pure compounds provided by Fluka, Merck, and Sigma-Aldrich, and quantification was performed by means of a calibration table built with the injection of standard solutions with known concentrations analyzed in wine samples (Vararu et al. 2016). The quantified compounds were identified and confirmed by GC–MS by an Agilent 7890 A with MSD-5975-C (Wilmington, DE, USA) using the same capillary column and programs for temperature and helium.

### Sensory analyses

Blind sensory analyses were carried out by a tasting panel of 9 judges, all tasters from the Department at the University of Córdoba. Randomized samples of 25–30 mL were served at 8–10 °C in clean and clear glasses with random letter labelling, and the aroma was preserved by covering the glasses with Petri dish lids. A descriptive test was conducted where the panel scored the wine visual, aromatic, and gustatory qualities on a scale of 0 to 100, with 100 representing the highest level of intensity and 0 signifying absence of intensity. Further, tasters were asked to indicate specific characters in each sensory phase (visual, olfactory, or taste).

### Statistical analysis

The significant differences between the various biological aging conditions were demonstrated by using Statgraphics v. XVI.I Multiple Variable Analysis (MVA) Principal Component Analysis (PCA) and multiple sample comparisons by homogeneous groups (HG), (StatPoint Technologies Inc., Warrenton, Virginia, USA).

## Results

### Ethanol and glycerol consumption

**Fig. 1:**
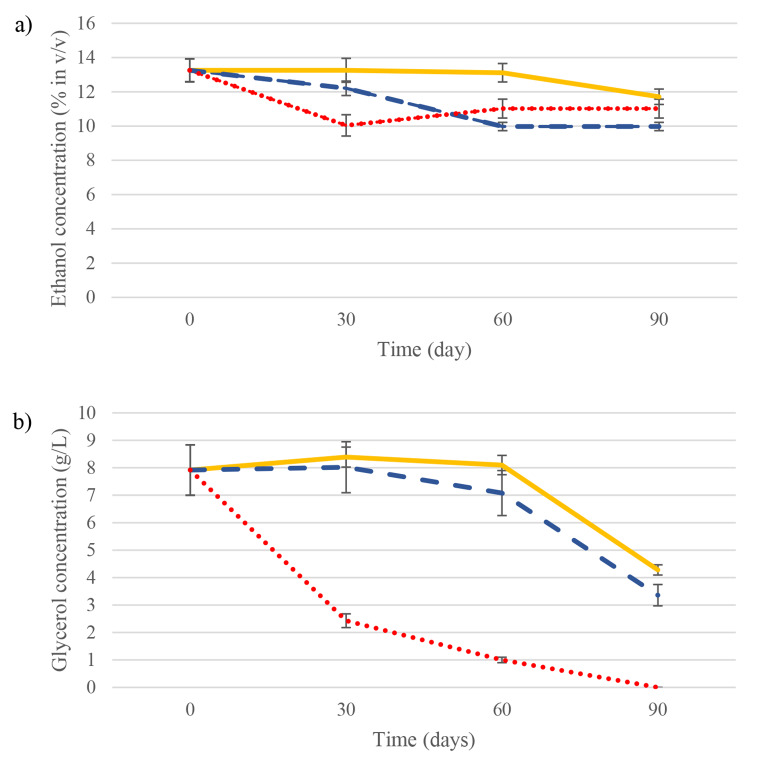
Ethanol percentage (v/v) (**a**) and glycerol (g/L) (**b**) quantified in the wine aged with microbial biocapsules (MB) in yellow line, the wine aged with free yeasts (FY) in blue dashed line, and the wine aged with free yeast without pre-acclimatization (NPFY) in red dots

Figure 1a shows total ethanol concentration decrease due to biological aging (not considering basal evaporation). Ethanol content dropped from 13.25 ± 0.67% (v/v) to 11.71 ± 0.45% (v/v) in MB wines while in FY wines, where pre-acclimatized flor yeast cells were not immobilized, ethanol decrease was more significant (from 13.25 ± 0.67% (v/v) to 9.97 ± 0.24% (v/v). Non-pre-acclimatized and non-immobilized flor yeasts (NPFY) consumed less ethanol than FY reaching a similar content than MB at day 90: from 13.25 ± 0.67% (v/v) to 11.02 ± 0.55% (v/v). As shown in Fig. 1b, in conditions using acclimatized yeasts, the glycerol concentration varied in a similar pattern between MB and FY: a slight increase in the first month (to 8.39 ± 0.36 g/L in MB and 8.02 ± 0.93 g/L in FY) and a drop from day 30 to 90 (to 4.28 ± 0.19 g/L in MB and 3.36 ± 0.39 g/L in FY). In NPFY the glycerol was mostly consumed during the first month (from 7.92 ± 0.17 to 2.43 ± 0.05 g/L). It is observed that ethanol and glycerol consumption by NPFY occurs mostly in the first month while in MB and FY agings, concentration of both metabolites decreased after this time.

### Acidity changes and major volatile compounds

The acidity related values are represented in Table 1. pH was slightly higher in all aged wines versus BW. The acidity, especially volatile acidity, was less in the biologically aged wines than in the BW. In MB and FY, the titratable acidity remained similar to BW while in NPFY it decreased to 4.10 ± 0.16 g H_2_T/L. Fixed acidity varied in a similar fashion than titratable acidity. The volatile acidity significantly decreased in all conditions, mostly in FY, while MB and NPFY had similar concentrations.

**Table 1:** Acidity parameters in base wine (BW) represented, the wine aged with microbial biocapsules (MB), the wine aged with free yeasts (FY), and the wine aged with free yeast without pre-acclimatization (NPFY). The different letters indicate homogeneous groups which significantly differ statistically in the parameters between the aging conditions (p < 0.05, F-test)

Acidity parameters	BW	MB	FY	NPFY
pH	3.25 ± 0.04^a^	3.46 ± 0.04^c^	3.32 ± 0.06^b^	3.45 ± 0.02^c^
Titratable acidity (g H_2_T/L)	5.05 ± 0.23^b^	5.13 ± 0.37^b^	5.26 ± 0.13^b^	4.10 ± 0.16^a^
Volatile acidity (g ACH/L)	0.42 ± 0.06^c^	0.20 ± 0.05^b^	0.11 ± 0.02^a^	0.20 ± 0.03^b^
Fixed acidity (g ACH/L)	4.63 ± 0.21^b^	4.78 ± 0.26^bc^	5.14 ± 0.20^c^	3.90 ± 0.18^a^

Major volatile compounds quantified in the aged wines are shown in Table 2. The 14 compounds found were the result of the yeast oxidative metabolism and they are only perceptible in smell if they exceed the perception threshold or OT (included in the table). Acetaldehyde, 3-methylbutanol, acetoin, 2-phenylethanol and ethyl-lactate were quantified over their OT in all conditions (Zea et al. 2007). The ethyl-acetate was reported above its OT in BW and MB wines while isobutanol in BW, and NPFY; and 2-3-butanediol (levo) in NPFY.

**Table 2:** Major volatile compounds concentration (mg/L) in base wine (BW), the wine aged with microbial biocapsules (MB), the wine aged with free yeasts (FY), and the wine aged with free yeast without pre-acclimatization (NPFY). The different letters indicate homogeneous groups which significantly differ statistically in the parameters between the aging conditions (p < 0.05, F-test). The odor threshold (OT) and the description of the smell/flavor taken from Zea et al. (2007), and CAS number are provided for each compound. N.f.: not found. The shaded cells show which concentrations are above the perception threshold of a specific compound

	CAS	OT (mg/L)	Odor/flavor description	BW	MB	FY	NPFY
Acetaldehyde (mg/L)	75-07-0	10	Over-ripe apple	122.33 ± 1.18^a^	450.18 ± 148.84^b^	121.31 ± 2.16^a^	415.18 ± 48.80^b^
Ethyl acetate (mg/L)	141-78-6	7.5	Pineapple, varnish, balsamic	82.1 ± 7.67^b^	7.77 ± 1.36^a^	-	-
1,1-Diethoxyethane (mg/L)	105-57-7		Refreshing, pleasant, fruity-green	-	14.37 ± 3.62^b^	4.91 ± 1.24^a^	13.45 ± 1.32^b^
Methanol (mg/L)	67-56-1	668	Chemical, medicinal	58.1 ± 14.82^b^	33.61 ± 2.43^a^	29.74 ± 5.09^a^	43.61 ± 5.88^a^
1-Propanol (mg/L)	71-23-8	830	Ripe fruit, alcohol	21.2 ± 0.44^b^	26.03 ± 1.44^c^	12.46 ± 1.09^a^	24.38 ± 2.17^c^
Isobutanol (mg/L)	78-83-1	40	Alcohol, wine like, nail polish	79.0 ± 1.13^d^	28.58 ± 0.50^b^	17.44 ± 4.49^a^	42.64 ± 3.24^c^
2-methylbutanol (mg/L)	137-32-6		Cooked roasted aroma with fruity or alcoholic undertones	86.7 ± 0.45^d^	35.64 ± 1.67^b^	23.29 ± 0.60^a^	51.51 ± 4.98^c^
3-methylbutanol (mg/L)	123-51-3	30	Disagreeable	460.18 ± 12.04^d^	205.22 ± 9.93^b^	146.28 ± 9.60^a^	257.90 ± 19.61^c^
Acetoin (mg/L)	53584-56-8	30	Buttery, creamy	45.43 ± 2.73^a^	291.91 ± 52.80^bc^	80.56 ± 2.53^ab^	401.63 ± 72.64^c^
Ethyl lactate (mg/L)	97-64-3	7,5	Strawberry, raspberry, buttery	120.71 ± 7.65^bc^	140.93 ± 5.42^ cd^	168.39 ± 39.24^d^	95.46 ± 10.68^ab^
2,3-butanediol (levo) (mg/L)	24347-58-8	668	Buttery, creamy	271.28 ± 37.51^a^	481.90 ± 38.54^b^	663.31 ± 14.99^c^	690.79 ± 22.58^d^
2,3-butanediol (meso) (mg/L)	5341-95-7	668	Buttery, creamy	98.09 ± 6.16^a^	160.13 ± 7.98^b^	202.45 ± 8.41^c^	308.99 ± 16.39^e^
Diethyl succinate (mg/L)	123-25-1	100	Over-ripe, lavender	14.46 ± 1.25^a^	18.68 ± 0.45^b^	22.90 ± 3.89^b^	20.31 ± 3.09^b^
2-phenylethanol (mg/L)	60-12-8	10	N.f	60.47 ± 6.25^a^	86.06 ± 7.56^b^	112.20 ± 15.89^c^	123.44 ± 15.83^c^

Acetaldehyde, the main product of biological aging, provides an over-ripe apple aroma. Its concentration reached similar levels in NPFY and MB while FY remained constant versus BW. A comparable trend was observed for 1,1-diethoxyethane concentration, which originates from acetaldehyde and contributes to fruity aromas and balsamic notes found in Sherry wines. Ethyl acetate (pineapple, varnish and balsamic aromas), isobutanol (alcohol, wine like and nail polish), and 3-methylbutanol (unpleasant) decreased in all aged wines while acetoin (buttery and creamy), 2,3-butanediol (levo) (buttery and creamy) and 2-phenylethanol (aroma description not found) increased. Ethyl lactate that provides strawberry, raspberry and buttery was quantified in lower quantities in wines aged with the pre-acclimatized yeasts (FY and MB).

### PCA analysis

**Fig. 2:**
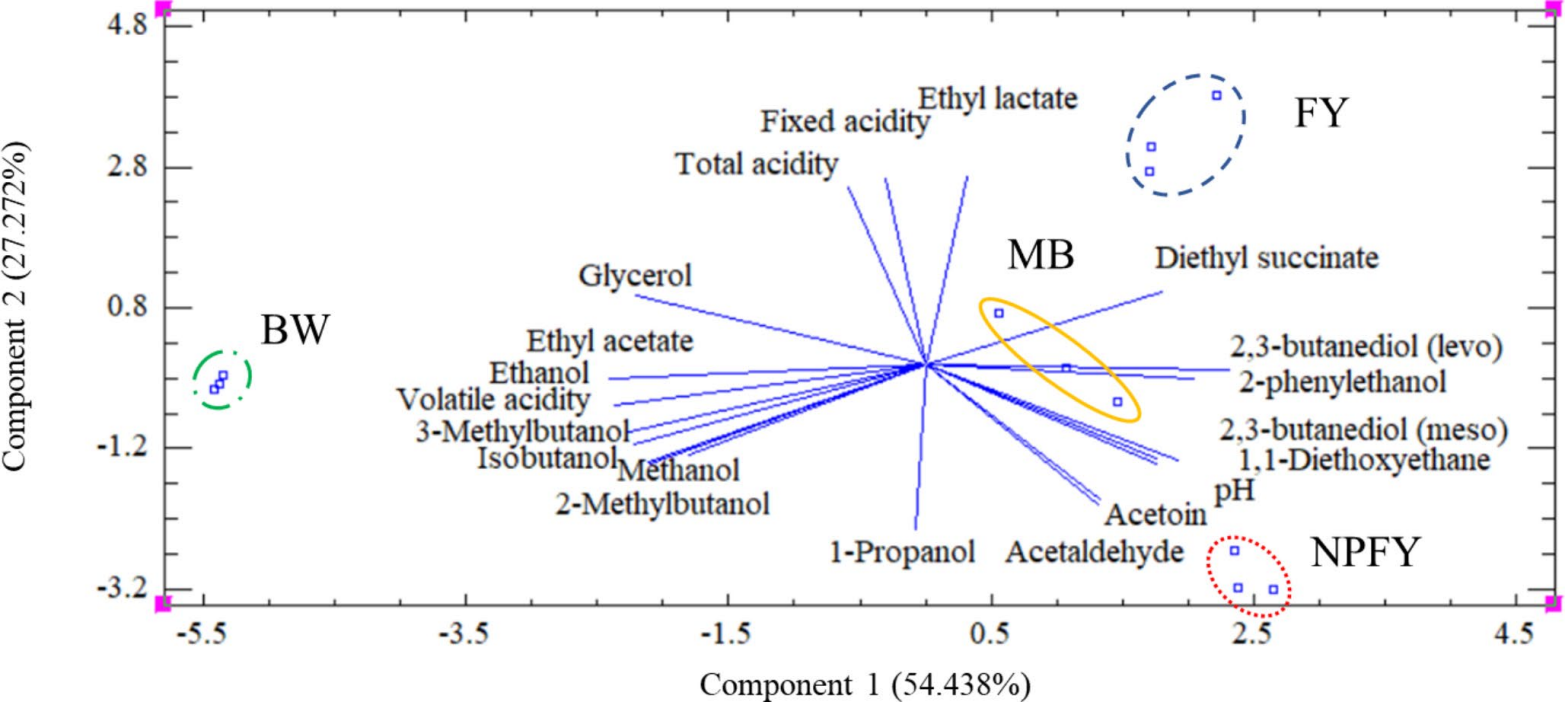
Principal component analysis (PCA) for chemical variables quantified in the base wine (BW) represented in green dot-dashed line, the wine aged with microbial biocapsules (MB) in yellow line, the wine aged with free yeasts (FY) in blue dashed line, and the wine aged with free yeast without pre-acclimatization (NPFY) in red dots

All chemical variables quantified were subjected to a PCA. PC1 accounted for 54.438% and PC2 accounted for 27.272% (Fig. 2). The unaged wine samples (BW) remained to the left of the plot while the aged wines were clearly separated on the right. Ethyl acetate, glycerol, total acidity, fixed acidity, ethyl lactate, diethyl succinate, 2,3-butanediol (levo) have positive projections over PC2 while the rest of the compounds, 2-phenylethanol, 2,3-butanediol (meso), 1,1-diethoxyethane, pH, acetoin, acetaldehyde, 1-propanol, 2-methylbutanol, methanol, isobutanol, 3-methylbutanol, volatile acidity, and ethanol have a negative projection. On the other hand, 1-propanol, 2-methylbutanol, methanol, isobutanol, 3-methylbutanol, volatile acidity, ethanol, ethyl acetate, glycerol, total acidity, fixed acidity have negative projections over PC1; and ethyl lactate, diethyl succinate, 2,3-butanediol (levo), 2-phenylethanol, 2,3-butanediol (meso), 1,1-diethoxyethane, pH, acetoin, and acetaldehyde have a positive projection. Diethyl succinate direct towards FY samples, also to MB samples along with 2,3-butanediol (levo), and 2-phenylethanol; pH, acetoin, and acetaldehyde project towards NPFY; and ethyl acetate, ethanol, and volatile acidity towards BW.

### Sensory analyses

**Fig. 3:**
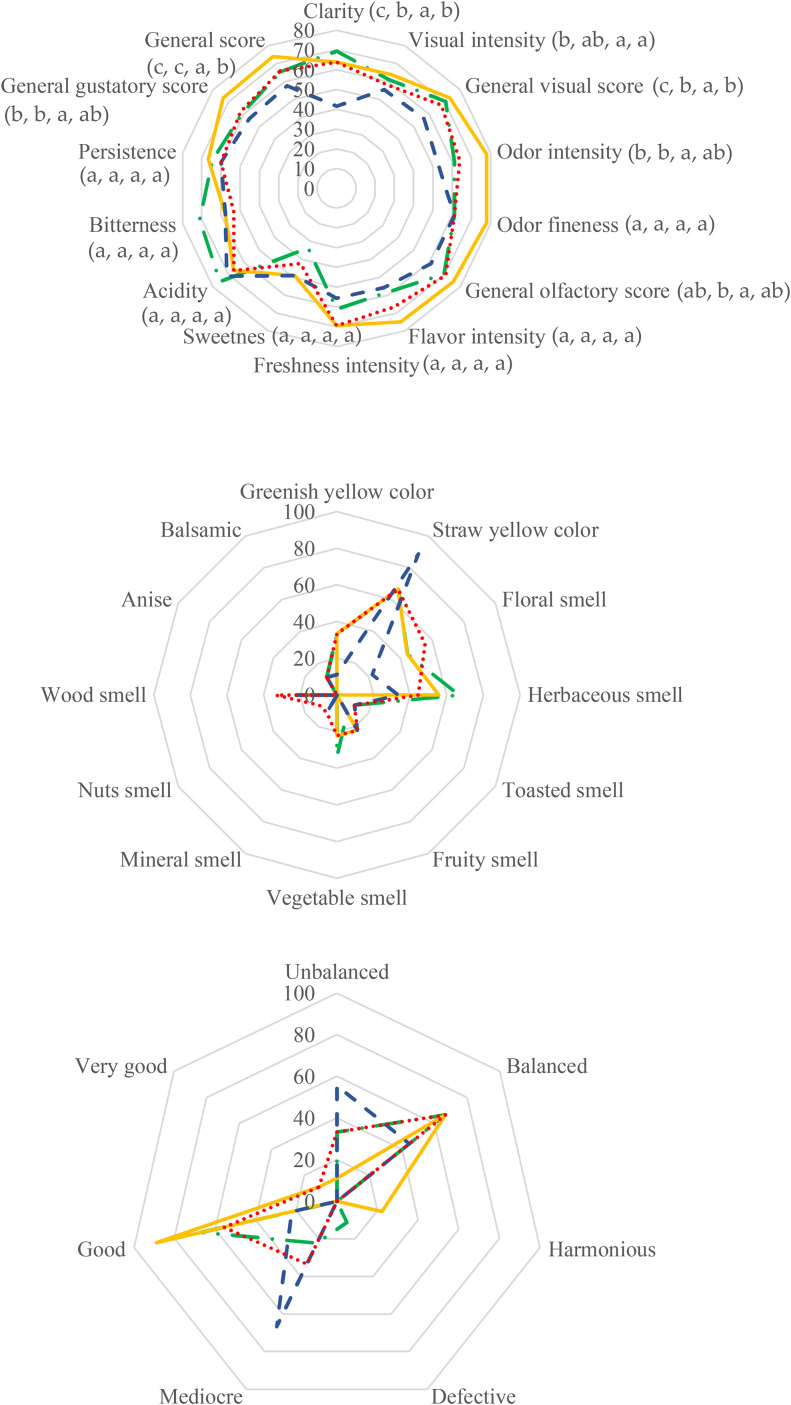
Sensory profile plot of the base wine (BW) represented in green dot-dashed line, the wine aged with microbial biocapsules (MB) in yellow line, the wine aged with free yeasts (FY) in blue dashed line, and the wine aged with free yeast without pre-acclimatization (NPFY) in red dots. Specific attributes are represented in a and b while general scores are represented in c. The different letters in parentheses in a indicate homogeneous groups (HG) (from left to right, the corresponding order is BW, MB, FY, and NPFY) which significantly differ statistically in the parameters between the aging conditions (p < 0.05, F-test). HG were not indicated in b and c as they represent frequencies of judges that perceived the different properties

The descriptive test showed that the wine with the highest quality score was MB among aged wines while FY wine had the lowest score (Fig. 3). Most of the tasters detected a pale straw yellow color in all wines and agreed that the most visually pleasant was the non-aged BW. Regarding odor, the highest intensity general olfactory scores were perceived in BW and MB wines. Floral and herbaceous smells were noticed in all wines by many tasters, especially in MB and NPFY. Tastewise, MB wines maintained their flavor intensity versus BW and were evaluated with the highest general score among the aged wines.

## Discussion

### Immobilization of flor yeast cells changes acidity and concentration of major volatile compounds

Immobilization of yeast cells results in changes in their physiology and metabolism (Norton and D’Amore 1994; Mallouchos et al. 2002; Moreno-García et al. 2018a). When using inert fungal supports as yeast cell carriers for alcoholic fermentations, increases in titratable acidity (or decreases), acetaldehyde, acetoin (or decreases), diacetyl and alcohols like isobutanol while decreases in volatile acidity have been reported (Peinado et al. 2005, 2006; Puig-Pujol et al. 2013; García-Martínez et al. 2015; López de Lerma et al. 2018; Ogawa et al. 2022). Here, we observed that immobilized flor yeast cells maintained titratable acidity while wines aged with the conventional method (NPFY) experienced a drop. Wines aged with FY showed a similar value to MB indicating that the difference between MB and NPFY is due to the pre-acclimatized to glycerol consumption rather than the immobilization effect. In white wines, this parameter is mostly dependent on the concentration of tartaric acid and other acids originated during alcoholic fermentation (i.e. succininc > malic > lactic acid, if they did not conduct malolactic fermentation). This decline could be due to the drop of concentration of some acids such as lactic, acetic, piruvic or gluconic acid during biological aging that are consumed as a carbon source by the yeast; or to their precipitation (e.g., tartaric acid into potassium bitartrate) (Pozo-Bayón et al. 2011). Yeast cells in MB or FY aging could be using other carbon sources aside from succinic, malic and/or lactic acid. Despite the drop in some values, these are within the typical values in white wine (4.00–8.00 g/L) (Rajkovic et al. 2007).

Volatile acidity decreased in a similar fashion in MB and NPFY. None of the analyzed wines overpassed 1.2 g/L which is the maximum allowed by the International Organization of Vine and Wine because higher concentrations lead to a vinegar-like flavor (Swiegers et al. 2005) and/or could indicate contamination by acetic acid bacteria (Bartowsky and Henschke 2008). Acetic acid is produced by the alcoholic fermentation at very low concentrations (no more than 0.7 g/L) and when the yeasts change to oxidative metabolism, acetic acid is consumed as a carbon source in the Krebs Cycle (Pozo-Bayón et al. 2011). A lower volatile acidity value in FY could be attributed to the unsuccessful pre-acclimatization step, which led yeast metabolism to prioritize consumption of acetic acid over glycerol.

**Fig. 4:**
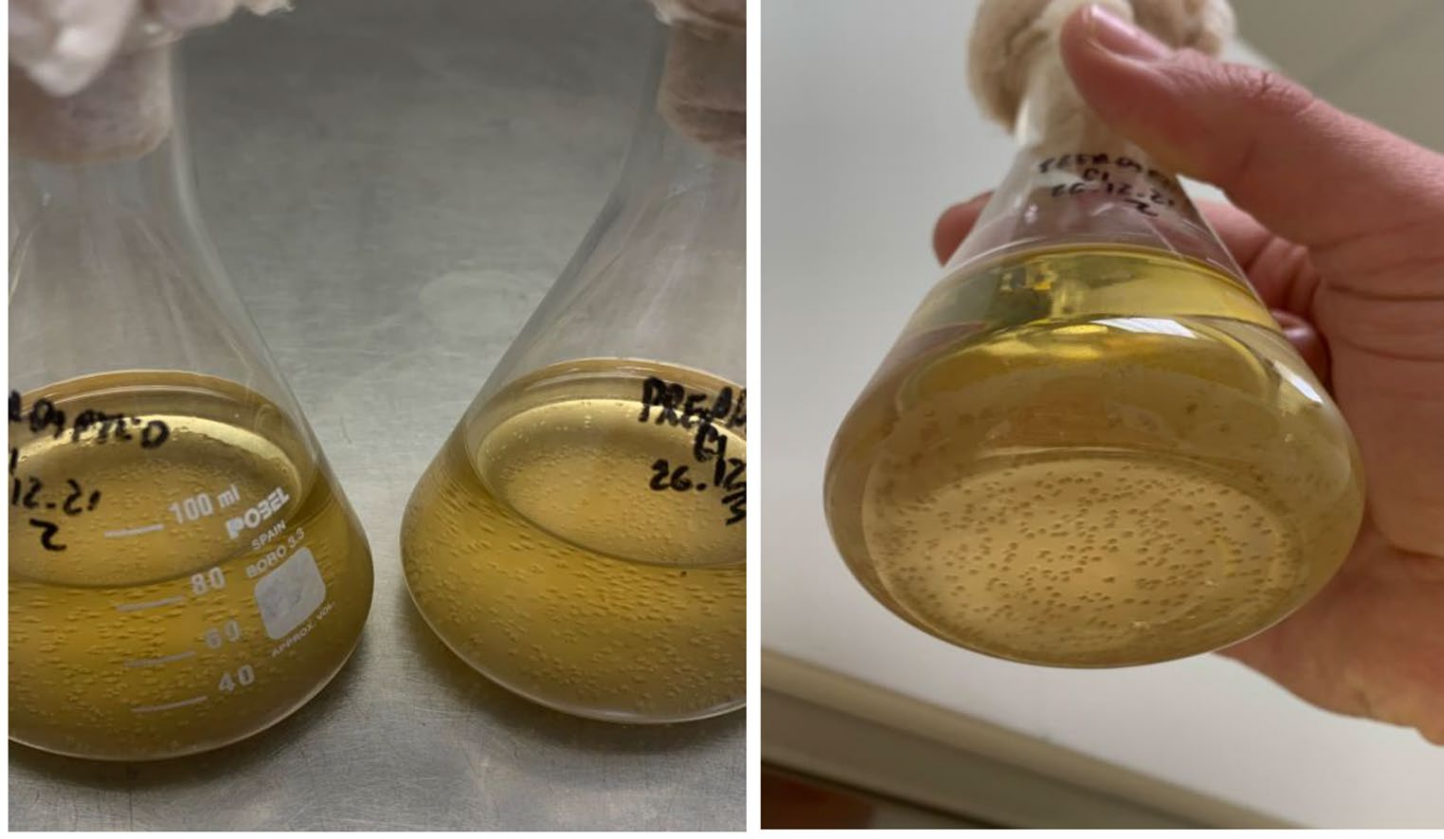
The pre-acclimatized free yeast (FY) growing at the bottom of the Erlenmeyer flask

Regarding the major volatile compounds, acetaldehyde is the most important compound in biological aging because it gives Sherry wines their characteristic aroma of ripe apples. Its concentration is usually found within 350–450 mg/L but sometimes can reach up to 1000 mg/L (Pozo-Bayón et al. 2011). It is produced when yeasts oxidize ethanol with alcohol dehydrogenase II (*ADH2*), which synthesis is suppressed by a high glucose concentration. As mentioned before, in all wines, acetaldehyde content reached the standard value except in FY which remained similar to BW. It must be noted that FY wine was the one with the lowest ethanol content indicating that its decrease is not a result of oxidation to acetaldehyde. Acetaldehyde synthesis depends on multiple factors such as the temperature and ethanol quantity but especially the oxygen availability and the redox potential. Zea et al. (2015) demonstrated that acetaldehyde is mostly produced when flor is formed. Here, we did not observe flor formation after three months of FY inoculation; instead, yeast cells precipitated to the bottom of the Erlenmeyer flask and formed viable colonies (Fig. 4). A low dissolved oxygen availability could explain the low ethanol oxidation into acetaldehyde in FY. An alternative or complementary hypothesis is that acetaldehyde was rapidly transformed into acetic acid by the aldehyde dehydrogenase (*ALD*) to use it as a carbon source (Pozo-Bayón et al. 2011; Moreno-García et al. 2015).

There is also shown that the pre-acclimatized yeast immobilized in fungal pellets promoted flor formation and acetaldehyde synthesis over the suspended yeast cells. Immobilization systems make cells immobilized more tolerant to stress conditions (i.e. high ethanol content) (Lapponi et al. 2022). A higher cell resistance can shorten the acclimatization phase or lag phase when inoculated in a new environment such as the BW, thus, shortening the onset of flor formation and acetaldehyde production.

### Flor yeast immobilization and glycerol pre-acclimatization do not significantly impact sensorial profile of aged wines

Although some chemical differences were reported among the wines tested, the wine tasting panel did not perceive significant differences in many of the sensory characters. This could be due to a masking effect of other aroma compounds present. Cell immobilization and glycerol pre-acclimatization did not significantly influence the wine sensorial profiles. Volatiles detected over their OT that are fruity aromas (acetaldehyde, ethyl acetate and ethyl lactate) were not clearly perceived among the tasters (10–25%). At low concentrations, the ethyl-acetate, in MB, produces a fruity pineapple smell, while at high concentrations as in BW, it has a balsamic smell.

Wines aged with MB reported the highest values in all categories (visual, aroma, and flavor) and general score among the aged wines while FY had the lowest in all these parameters. These results indicate that under the studied conditions, cell immobilization improves the sensorial attributes over suspended yeast cells both pre-acclimatized to glycerol consumption or not (conventional technique). Higher sensorial qualities have also been reported when using biocapsules to produce sparkling wine, long aged sparkling wine, and IPA beer (Puig-Pujol et al. 2013; García-Martínez et al. 2015; López de Lerma et al. 2018).

### Glycerol consumption was unsuccessful in pre-acclimatized yeast cells

In the absence of fermentable carbon sources, *S. cerevisiae* catabolizes ethanol, glycerol, and acetic acid as carbon sources during biological aging in order to synthesize hexoses and polysaccharides through gluconeogenesis or to produce energy through the Krebs Cycle, electron transport chain, and ATP synthesis (Mauricio et al. 1997). In this work, the wine aged using flor yeasts pre-acclimatized and immobilized in fungal pellets (MB) maintained a significantly higher ethanol content throughout the aging procedure while not consuming high amounts of glycerol. Furthermore, compared to non-pre-acclimatized free flor yeast (NPFY), unexpectedly low glycerol consumption rates and high ethanol consumption rates were observed in the pre-acclimatized flor yeasts (FY).

These results may be due to an unsuccessful glycerol pre-acclimatization where flor yeast did not activate the glycerol metabolic routes. As set out above, glycerol is a compound consumed during biological aging so it can be used instead or in combination with ethanol (Pozo-Bayón and Moreno-Arribas 2016). Growth of *S. cerevisiae* strains on glycerol is very low, with a reported growth rate of 0.05 h − 1 (Lu et al. 2005). This means that the flor yeast during the glycerol pre-acclimatization may still be in their lag phase at day 5 in the YPG culture and in the 9 g/L glycerol BW and yeast viability could be low leading to longer lag phases during biological aging (slighter changes in ethanol and glycerol concentrations during the first months as observed in Figs. 1 and 2). In the published literature, there is very little information about the maximum growth rate and biomass output of *S. cerevisiae* grown on glycerol as the only carbon source. (Lages and Lucas 1997) reported after 3 days of lag phase a maximal growth rate of 0.1 h − 1 for a *S. cerevisiae* strain grown on a mineral medium containing glycerol, peptone and yeast extract as carbon sources.

Some authors investigated techniques to promote yeast growth on glycerol. (Eugene Raj et al. 2002) used a culture medium containing glycerol, yeast extract, and glucose with vitamin and trace elements supplementation in a fed-batch bioreactor and reported growth rates of 0.35 h and attained a 20-fold growth rate (from 0.01 to 0.2 h − 1) through sequential sub-cultivations. These authors repeatedly transferred the yeast 20 times into fresh mineral medium (pH 4) containing 20 g/L glycerol as the sole carbon source and cultured at 30 °C on a rotary shaker (160 rpm) overnight. For each transfer, cells at the middle of their exponential phase were used to start the next sub-cultivation with fresh medium (10% v/v inoculation rate). The μmax increased during the first 50 cell generations (from 0.01 to 0.2 h − 1).

Considering the factors mentioned above, it is possible to enhance the growth of yeast cells on glycerol by utilizing lower concentrations, a vitamin supplement, a fed-batch bioreactor culture mode, and/or performing sequential yeast sub-cultivations. In addition, triggering biofilm formation under static conditions may also be beneficial as it can prepare the yeasts physiologically for the base wine inoculum.

## Conclusion

In this work, we produced for the first time Sherry wine using an immobilization methodology, so-called microbial biocapsules, where flor yeast cells are entrapped in inactivated pellets of *A. oryzae*, a food-grade filamentous fungus generally recognized as safe. Chemical differences were reported when compared to a Sherry wine elaborated with suspended yeast cells (the conventional method) and significantly higher scores were given after the sensorial analysis to those wines produced with microbial biocapsules. Furthermore, before immobilization, flor yeasts have been pre-acclimatized for glycerol consumption to reduce ethanol catabolism with the final goal of avoiding or complementing the fortification step. Although the methodology used in this work was not successful, optimization in the pre-acclimatization step was suggested: using lower glycerol concentrations in the medium, a vitamin supplement, a fed-batch bioreactor culture mode, performing sequential yeast sub-cultivations, and/or use of static acclimatization so that the yeast forms flor and does not have to re-acclimatized when inoculated. Nonetheless, the immobilization of these cells reduced the ethanol consumption during aging which could be useful to maintain ethanol levels above 14% (v/v) thus, decreasing the ethanol volumes required in fortification. Taking into account that immobilization systems facilitate yeast handling and recovery, increase protection of cells from stress conditions, reduce ethanol consumption during aging, and improve wine sensorial characters; we believe that immobilization of flor yeasts in microbial biocapsules may be an economic technique which produces high quality differentiated wines. This combined with an optimized pre-acclimatization to glycerol consumption could be regarded as an interesting technique for Sherry winemaking. Further experiments at barrel scale and techno-economical analysis should be conducted in order to confirm the potential of this methodology.

## Data Availability

All data generated or analyzed during this study are included in this published article.

## Declarations

### Patents

The microbial biocapsule assembling methodology has been submitted to patent application (Application number: 64/411,843).
